# Altered thiol/disulfide homeostasis in patients with diabetes mellitus and its chronic complications

**DOI:** 10.20945/2359-4292-2025-0042

**Published:** 2025-09-22

**Authors:** Nuri Aslanoğlu, Şakir Özgür Keşkek, Salim Neşelioğlu, Funda Eren

**Affiliations:** 1 Department of Internal Medicine, Adana City Training and Research Hospital, Adana, Turkey; 2 Department of Internal Medicine, Alanya Alaaddin Keykubat University, Alanya, Turkey; 3 Department of Biochemistry, Ankara Yıldırım Beyazıt University, Faculty of Medicine, Ankara, Turkey; 4 Department of Biochemistry, Ankara City Hospital, Ankara, Turkey

**Keywords:** Disulfides, Homeostasis, Diabetic angiopathies, Prediabetic state, Biomarkers

## Abstract

**Objective:**

To evaluate the effect of diabetes mellitus and its chronic complications on thiol/disulfide homeostasis.

**Methods:**

The study included 381 participants divided into six groups: healthy controls (Group 1; n = 91), patients with prediabetes (Group 2; n = 50), patients with diabetes mellitus without complications (Group 3; n = 70), patients with diabetic retinopathy (Group 4; n = 47), patients with diabetic nephropathy (Group 5; n = 70), and patients with diabetic foot (Group 6; n = 53). Thiol/disulfide homeostasis was determined by measuring the reduction reaction of oxidized thiols.

**Results:**

Native thiol levels were low in patients with diabetes mellitus complications (Group 4, 264.7 ± 58.5 µmol/L; Group 5, 246.6 ± 67.5 µmol/L; Group 6, 174.3 ± 65.9 µmol/L), as were total thiol levels. The highest and lowest disulfide levels were observed in Group 1 (controls; 20.4 ± 5.2 µmol/L) and Group 6 (16.2 ± 5.7 µmol/L), respectively. The disulfide/native thiol ratio was increased in Groups 4, 5, and 6 compared with Groups 1, 2, and 3.

**Conclusion:**

The presence of diabetes mellitus complications substantially decreased native thiol, total thiol, and disulfide levels.

## INTRODUCTION

The worldwide prevalence of diabetes mellitus (DM) has been rising and is becoming an important public health concern (^1^). Notably, DM is a complicated metabolic disease characterized by impaired glucose and insulin homeostasis. The development of microvascular DM complications - nephropathy, retinopathy, and neuropathy - is associated with hyperglycemia, hyperlipidemia, epigenetic dysregulation, and genetic factors (^2^). It is also associated with oxidative stress, an imbalance between oxidants and antioxidants (^3^) that is known to be a primary risk factor with a crucial role in the initiation and progression of DM (^4^). Chronic oxidative stress leads to cellular damage, inflammatory responses, and endothelial dysfunction - all key factors in the pathogenesis of diabetic complications.

Dynamic thiol/disulfide homeostasis is crucial for antioxidant defense and contributes to critical processes such as signal reduction, apoptosis, and enzyme activity regulation (^5^). Several intrinsic and extrinsic factors may affect thiol/disulfide homeostasis, including age, sex, diet, smoking, inflammation, aging, lifestyle, use of medications, air pollution, and radiation (^6^). Thiols are important antioxidant molecules that help eliminate reactive oxygen species. Reversible disulfide bonds are formed by the oxidation of thiols with reactive oxygen species. These disulfide bonds may be reduced to thiols to maintain dynamic thiol/disulfide homeostasis (^7^). Thus, thiol/disulfide homeostasis serves as a sensitive indicator of oxidative stress levels, providing important insights into redox regulation in diseases such as DM.

The increased production of reactive oxygen species during hyperglycemia plays a critical role in the pathogenesis of DM complications (^8^). Previous studies have shown that oxidative stress levels are increased in patients with prediabetes (^9^), DM, and DM complications such as nephropathy (^10^), retinopathy (^11^), and diabetic foot (^12^). However, none of these studies compared the impacts of different microvascular or macrovascular complications on the thiol/disulfide homeostasis within the same study. Therefore, filling this gap will contribute to a deeper understanding of the association between oxidative stress and the development of DM complications. We hypothesized that patients with DM, particularly those with chronic complications, exhibit significant alterations in thiol/disulfide homeostasis compared with healthy individuals. Based on this hypothesis, the aim of this study was to evaluate the effect of DM and its chronic complications on thiol/disulfide homeostasis.

## METHODS

### Study design and patient selection

This case-control study was conducted at the Internal Medicine Clinic of Adana City Training and Research Hospital between September 2017 and May 2018. The study protocol was approved by the Ethics Committee of Adana City Training and Research Hospital (Adana, Turkey; decision number 103, dated September 13, 2017) and was conducted in accordance with the principles outlined in the Declaration of Helsinki. All participants signed a written informed consent.

Patients with chronic diseases other than DM were excluded from the study. A power analysis (80% power, 0.05 type 1 error) was performed to determine the optimal sample size for the study. A total of 381 participants were included. The control group consisted of 91 healthy subjects (Group 1; n = 91). The remaining 290 participants were divided into five groups, namely, prediabetes (Group 2; n = 50), DM without complications (Group 3; n = 70), diabetic retinopathy (Group 4; n = 47), diabetic nephropathy (Group 5; n = 70), and diabetic foot (Group 6; n = 53). Among the 170 participants included in Groups 4, 5, and 6, 40% had multiple complications. Group 4 included patients with retinopathy. Of those with mixed complications, 23 patients had all three complications (retinopathy, nephropathy, and diabetic foot), while 9 patients with both retinopathy and diabetic foot were assigned to Group 6. Additionally, 36 patients with both nephropathy and retinopathy were included in Group 5.

The oral glucose tolerance test (OGTT) and measurement of glycated hemoglobin (HbA1c) levels were used to diagnose prediabetes. Patients with fasting glucose levels of 100 to 125 mg/dL, 2-hour postprandial glucose levels of 140 to 199 mg/dL, and HbA1c levels of 5.7 to 6.4% were included in the prediabetes group. Nephropathy was determined in the presence of microalbuminuria or macroalbuminuria. Patients with an albumin/creatinine ratio of 30 to 300 mg/g (microalbuminuria) or > 300 mg/g (macroalbuminuria) were included in the nephropathy group. Retinopathy was diagnosed by an ophthalmologist. The skin of the participants’ feet was carefully examined, including the spaces between the toes, and the patients with foot ulcers were allocated to the diabetic foot group. Body mass index was calculated by dividing body weight (kg) by the square of height (m^2^).

### Biochemical analysis

Levels of HbA1c were measured using the Premier Hb9210 analyzer (Trinity Biotech, Ireland). Levels of fasting plasma glucose (FPG), low-density lipoprotein (LDL) cholesterol, triglycerides, high-density lipoprotein (HDL) cholesterol, serum creatinine, aspartate aminotransferase (AST), and alanine transaminase (ALT), along with albumin and creatinine in spot urine, were recorded from previous analyses, where they were measured using the Beckman Coulter AU5800 analyzer (Beckman Coulter Inc., Brea, CA, USA).

### Thiol/disulfide homeostasis

Thiol/disulfide homeostasis was determined using a spectrophotometric method, as described by Erel and cols. (^13^). First, 2 mL of venous blood samples were drawn from all participants and centrifuged at 4,000 rpm for 10 minutes to separate plasma and serum. After centrifugation, the serum samples were immediately stored in Eppendorf tubes at -80 ºC until the analysis. In this method, the total thiol was detected after treatment with sodium borohydride (NaBH4) to reduce the oxidized thiols. Formaldehyde was used to remove any unused NaBH4 from the system. Subsequently, the native and total thiol amounts in the samples were detected after reacting with 5,5’-dithiobis-(2-nitrobenzoic) acid. The dynamic disulfide amount (oxidized thiol) was calculated as half the difference between total thiol and native thiol. The ratios of native thiol/total thiol (%), disulfide/native thiol (%), and disulfide/total thiol (%) were also calculated.

### Statistical analysis

The statistical analysis was performed using MedCalc, version 18.11.3 (MedCalc Software, Ostend, Belgium). The normality of the data was assessed using the Kolmogorov-Smirnov test. The Chi-squared was used to compare categorical variables between groups. For comparisons of quantitative variables among more than two groups, either analysis of variance (Anova) or the Kruskal-Wallis test was applied, depending on the distribution of the data. Pearson correlation coefficient (r), along with p values and 95% confidence intervals, were used to assess the strength and direction of associations between continuous variables. Multiple regression analysis using the backward elimination method was performed to evaluate the relationship between a dependent variable and one or more independent (predictor or explanatory) variables. The probability of a type I error (alpha) was set at 0.05 in all tests.

## RESULTS

As shown in **Table 1**, no significant differences in age (p = 0.250), sex (p = 0.369), or body mass index (BMI) (p = 0.914) were observed among the groups. The prevalence of female participants ranged from 48 to 68.1% across the groups, while age varied between 48.4 ± 10.4 years and 51.8 ± 10.5 years. The BMI of the participants in all groups fell outside the healthy range (18.5 to 24.9 kg/m^2^).

**Table 1:** Demographic characteristics and laboratory results

	Group 1 (n = 91)	Group 2 (n = 50)	Group 3 (n = 70)	Group 4 (n = 47)	Group 5 (n = 70)	Group 6 (n = 53)	p-values^*^
Sex, female	59 (64.8%)	24 (48%)	43 (61.4%)	32 (68.1%)	45 (64.3%)	32 (60.4%)	0.369
Age, years	49.4 ± 8.7	48.4 ± 10.4	50.0 ± 8.4	50.7 ± 5.6	51.8 ± 10.5	51.7 ± 6.4	0.250
BMI, kg/m^2^	26.7 ± 6.1	32.9 ± 7.6	32.2 ± 6.1	32.8 ± 6.0	32.0 ± 5.6	28.7 ± 5.9	0.914
HbA1c, %	5.3 ± 0.29	6.0 ± 0.3	8.9 ± 2.27	9.7 ± 2.49	9.8 ± 2.57	10.5 ± 2.33	0.001
HbA1c, mmol/mol	34.8 ± 4.8	42.2 ± 2.8	74.0 ± 25.1	82.7 ± 27.1	83.7 ± 28.0	92.3 ± 25.7	0.001
FPG, mg/dL	91.9 ± 8.6	105.9 ± 19.6	201.3 ± 93.2	235.5 ± 111.2	234.2 ± 110.7	301.0 ± 136.5	0.001
LDL cholesterol, mg/dL	116.6 ± 23.9	129.3 ± 51.9	125.9 ± 36.5	130.3 ± 44.5	136.6 ± 54.4	102.4 ± 36.3	0.001
Triglycerides, mg/dL	122.3 ± 60.7	173.4 ± 82.6	220.2 ± 197.6	223.5 ± 149.5	248.5 ± 188.2	196.0 ± 124.8	0.0001
HDL cholesterol, mg/dL	50.5 ± 11.7	46.3 ± 14.3	45.3 ± 8.4	39.4 ± 18.6	49.7 ± 16.4	34.9 ± 10.8	0.001
Creatinine, mg/dL	0.7 ± 0.1	0.7 ± 0.1	0.6 ± 0.2	0.7 ± 0.2	1.0 ± 0.8	1.3 ± 1.2	0.001
AST, U/L	19.6 ± 4.7	23.8 ± 5.8	23.3 ± 10.8	20.5 ± 8.9	20.9 ± 6.6	20.5 ± 29.7	< 0.0001
ALT, U/L	18.0 ± 8.7	24.9 ± 13.8	28.6 ± 18.0	21.7 ± 12.6	21.3 ± 10.3	16.9 ± 24.4	< 0.00001

* Comparison among all groups.

Results expressed as n (%) or mean ± standard deviation.

Group 1: healthy control subjects; Group 2: individuals with prediabetes; Group 3: patients with diabetes mellitus without complications; Group 4: patients with diabetic retinopathy; Group 5: patients with diabetic nephropathy; Group 6: patients with diabetic foot ulcers.

BMI: body mass index; HbA1c: glycated hemoglobin; FPG: fasting plasma glucose; LDL: low-density lipoprotein; HDL: high-density lipoprotein; AST: aspartate aminotransferase; ALT: alanine transaminase.

Levels of HbA1c were significantly higher in the groups of DM without complications and DM with complications compared with Groups 1 (controls) and 2 (individuals with prediabetes; p = 0.001). Additionally, a significant difference was observed between groups regarding levels of FPG (p = 0.001), LDL cholesterol (p = 0.001), cholesterol (p = 0.001), triglycerides (p = 0.0001), and HDL cholesterol (p = 0.001). Creatinine levels were comparable in Groups 1, 2, 3, and 4 and were increased in the groups with diabetic nephropathy and diabetic foot. Creatinine, AST, and ALT levels differed significantly among the groups (p = 0.001, p < 0.0001, and p < 0.00001, respectively).

**Table 2** presents the thiol/disulfide homeostasis parameters used to estimate oxidative stress levels. There was a significant difference in native thiol (p = 0.016), total thiol (p = 0.004), and disulfide levels (p = 0.029) across the groups. The highest native thiol, total thiol, and disulfide levels were measured in Group 1 (controls), followed, in order, by Groups 3, 2, 4, 5, and 6. Opposed to this trend, the highest and lowest disulfide/native thiol levels (%) were observed in Groups 6 and 1, respectively. Furthermore, a significant difference was observed in the ratios of disulfide/native thiol (p < 0.001), disulfide/total thiol (p = 0.001), and native thiol/total thiol across the groups (p = 0.001).

**Table 2:** Comparison of thiol/disulfide homeostasis parameters among groups

	Group 1	Group 2	Group 3	Group 4	Group 5	Group 6	p-values^*^
Native thiol, µmol/L	331.3 ± 48.2	295.9 ± 51.5	300.6 ± 52.2	264.7 ± 58.5	246.6 ± 67.5	174.3 ± 65.9	0.016
Total thiol, µmol/L	372.3 ± 49.0	334.1 ± 51.7	338.0 ± 50.2	303.9 ± 57.4	282.5 ± 71.0	201.8 ± 67.1	0.004
Disulfide, µmol/L	20.4 ± 5.2	19.0 ± 6.1	18.7 ± 5.3	18.7 ± 3.7	18.5 ± 6.5	16.2 ± 5.7	0.029
Disulfide/native thiol, %	6.35 ± 2.07	6.7 ± 2.9	6.6 ± 3.2	7.4 ± 2.2	8.2 ± 4.2	10.4 ± 4.3	<0.001
Disulfide/total thiol, %	5.6 ± 1.6	5.8 ± 2.1	5.7 ± 2.2	6.4 ± 1.6	6.9 ± 2.7	6.4 ± 2.7	0.001
Native thiol/total thiol, %	88.8 ± 3.1	88.3 ± 4.2	88.5 ± 4.3	87.2 ± 3.2	86.2 ± 5.5	83.1 ± 5.5	0.001

* Comparison among all groups.

Group 1: healthy control subjects; Group 2: individuals with prediabetes; Group 3: patients with diabetes mellitus without complications; Group 4: patients with diabetic retinopathy; Group 5: patients with diabetic nephropathy; Group 6: patients with diabetic foot ulcers.

One of the goals of this study was to evaluate the effect of DM complications, regardless of the type, on biochemical parameters and thiol/disulfide homeostasis. To achieve this, we combined the prediabetes and DM without complications groups (Groups 2 and 3) into a single group and did the same for the DM complications groups (Groups 4, 5, and 6). Both new groups were then compared against the control group (Group 1). We found no significant differences between the groups in terms of sex (p = 0.387) or age (p = 0.181; **Table 3**). However, BMI values differed significantly (p < 0.0001). In the groups with DM complications (Group 4+5+6), HbA1c values were significantly higher compared with the groups with prediabetes and DM without complications (Group 2+3). Similarly, FPG levels were higher in the combined groups with DM (Group 4+5+6) compared with Group 2+3, while levels of LDL cholesterol (p = 0.003), triglycerides (p < 0.0001), HDL cholesterol (p = 0.00003), creatinine (p < 0.0001), AST (p < 0.0001), and ALT (p < 0.0001) also differed significantly between these groups.

**Table 3:** Demographic characteristics and laboratory results in the combined groups

	Group 1 (n = 91)	Group 2+3 (n = 120)	Group 4+5+6 (n = 170)	p-value^*^
Sex, female	59 (64.8%)	67 (55.8%)	98 (57.6%)	0.387
Age, years	49.4 ± 8.7	49.3 ± 9.3	51.5 ± 8.1	0.181
BMI, kg/m^2^	26.7 ± 6.1	32.5 ± 6.7	31.2 ± 6.0	< 0.0001
HbA1c, %	5.3 ± 0.3	7.7 ± 2.2	10.0 ± 2.4	< 0.0001
HbA1c, mmol/mol	34.8 ± 4.7	60.7 ± 24.8	86.1 ± 27.2	< 0.0001
FPG, mg/dL	91.9 ± 8.6	162.9 ± 85.2	255.4 ± 122.7	< 0.001
LDL cholesterol, mg/dL	116.6 ± 23.9	131.7 ± 36.8	126.4 ± 46.2	0.003
Triglycerides, mg/dL	122.3 ± 60.7	201.9 ± 163.7	225.0 ± 160.1	< 0.0001
HDL cholesterol, mg/dL	50.5 ± 11.7	45.7 ± 11.0	42.1 ± 16.7	0.00003
Creatinine, mg/dL	0.7 ± 0.1	0.7 ± 0.1	1.2 ± 0.9	< 0.0001
AST, U/L	19.6 ± 4.7	23.5 ± 9.1	21.0 ± 17.7	< 0.0001
ALT, U/L	18.0 ± 8.7	27.1 ± 16.5	20.0 ± 16.5	< 0.0001

* Comparison among all groups.

Results expressed as n (%) or mean ± standard deviation.

Group 1: healthy control subjects; Group 2: individuals with prediabetes; Group 3: patients with diabetes mellitus without complications; Group 4: patients with diabetic retinopathy; Group 5: patients with diabetic nephropathy; Group 6: patients with diabetic foot ulcers.

BMI: body mass index; HbA1c: glycated hemoglobin; FPG: fasting plasma glucose; LDL: low-density lipoprotein; HDL: high-density lipoprotein; AST: aspartate aminotransferase; ALT: alanine transaminase.

The oxidative stress level observed in Group 2+3 differed from that in Group 4+5+6, in which there was a higher reduction in both native and total thiol compared with Group 1 and Group 2+3 (**Table 4**). Concurrently, the disulfide amount decreased while the ratios of disulfide/native thiol or disulfide/total thiol increased significantly in Group 4+5+6 relative to the other groups (p < 0.0001 and p = 0.00001, respectively). Ratios of disulfide/native thiol, disulfide/total thiol, and native thiol/total thiol were comparable in Group 1 and Group 2+3.

**Table 4:** Comparison of thiol/disulfide homeostasis parameters among combined groups

	Group 1 (n = 91)	Group 2+3 (n = 120)	Group 4+5+6 (n = 170)	p-value^*^
Native thiol, µmol/L	331.3 ± 48.2	298.6 ± 51.8	229.0 ± 74.5	0.001
Total thiol, µmol/L	372.3 ± 49.0	336.4 ± 50.6	263.2 ± 78.3	0.001
Disulfide, µmol/L	20.4 ± 5.2	18.8 ± 5.6	17.8 ± 5.7	0.0003
Disulfide/native thiol, %	6.4 ± 2.0	6.6 ± 3.0	8.6 ± 3.9	< 0.0001
Disulfide/total thiol, %	5.6 ± 1.6	5.7 ± 2.1	7.2 ± 2.6	< 0.00001
Native thiol/total thiol, %	88.8 ± 3.1	88.5 ± 4.2	85.5 ± 5.2	< 0.00001

* Comparison among all groups.

Group 1: healthy control subjects; Group 2: individuals with prediabetes; Group 3: patients with diabetes mellitus without complications; Group 4: patients with diabetic retinopathy; Group 5: patients with diabetic nephropathy; Group 6: patients with diabetic foot ulcers.

**Table 5** shows the results of correlation analyses between native thiol, total thiol, and disulfide levels in relation to age, BMI, DM duration, as well as FPG and HbA1c levels. The strongest negative correlation was observed between total thiol and DM duration (r=-0.607; p < 0.0001).

**Table 5:** Pearson correlation analysis of native thiol, total thiol, and disulfide levels in relation to participants’ characteristics

	Native thiol	Total thiol	Disulfide
Age	r = -0.250 p < 0.0001	r = -0.262 p < 0.0001	r = -0.08 p = 0.116
BMI	r = -0.123 p = 0.016	r = -0.097 p = 0.05	r = 0.027 p = 0.591
Diabetes duration	r = -0.571 p < 0.0001	r = -0.607 p < 0.0001	r = -0.218 p < 0.0001
FPG	r = -0.429 p < 0.001	r = -0.438 p < 0.001	r = -0.211 p < 0.001
HbA1c	r = -0.479 p < 0.001	r = -0.463 p < 0.001	r = -0.169 p = 0.0009

BMI: body mass index; FPG: fasting plasma glucose; HbA1c: glycated hemoglobin.

Multiple regression analysis was performed with total thiol as the dependent variable and FPG, BMI, HbA1c, age, and DM duration, as well as the presence of nephropathy, retinopathy, and diabetic foot ulcer, as independent variables (**Table 6**). A significant correlation remained between total thiol and BMI value (p = 0.0004), HbA1c level (p = 0.0004), DM duration (p < 0.0001), age (p < 0.0001), nephropathy (p = 0.0002), and diabetic foot ulcers (p < 0.0001).

**Table 6:** Results of multiple regression analysis, with total thiol as the dependent variable

Independent variables	Coefficient	Standard error	t	p-values
Constant	517.3170			
BMI, kg/m^2^	-1.5832	0.4398	-3.600	0.0004
HbA1c, %	-4.2072	1.2604	-3.338	0.0009
Diabetes duration, years	-2.2989	0.5244	-4.384	< 0.0001
Age, years	-1.7561	0.3350	-5.241	< 0.0001
Diabetic foot	-80.9199	9.2986	-8.702	< 0.0001
Nephropathy	-29.5381	7.7047	-3.834	0.0002

BMI: body mass index; HbA1c: glycated hemoglobin.

## DISCUSSION

In the present study, we investigated alterations in thiol/disulfide homeostasis in patients with prediabetes, DM, and DM complications (retinopathy, nephropathy, and diabetic foot). The highest levels of native thiol, total thiol, and disulfide were detected in Group 1 (controls), whereas the lowest levels were measured in Group 6. Conversely, the lowest and highest disulfide/native thiol ratios were detected in Group 1 and Group 6, respectively.

The native thiol and total thiol levels were higher in Group 3 compared with Group 2. While this outcome was unexpected, it became less surprising upon considering the more intensive medical treatment that Group 3 received. We suspect that the treatment administered to the patients in Group 3 might have indirectly mitigated the oxidative stress they exhibited.

Partially supporting our findings, lower native and total thiol levels in patients with prediabetes compared with controls have also been reported by Ates and cols. (^14^), although their study included patients with higher disulfide levels than ours. The authors also found that the higher disulfide levels and greater disulfide/native thiol and disulfide/total thiol ratios were linked to higher oxidative stress and moderate hyperglycemia in patients with prediabetes compared with controls (^14^). The ratios of native thiol/total thiol, disulfide/native thiol, and disulfide/total thiol are important indicators of dynamic thiol/disulfide homeostatic status; these parameters help estimate the presence of oxidative stress, enabling earlier intervention to identify patients at higher risk of developing complications. Both non-proliferative and proliferative diabetic retinopathy have been associated with decreased native and total thiol compared with the absence of diabetic retinopathy (^15^), which is in agreement with our result. Similar to the study by Ates and cols. (^14^), higher levels of disulfide, disulfide/native thiol, and disulfide/total thiol have also been observed in proliferative compared with non-proliferative diabetic retinopathy, and DM without retinopathy (^15^). In a study including patients with diabetic nephropathy, native and total thiol levels decreased, while disulfide levels increased with increasing albuminuria across all groups (^10^). All these studies showed similar results using the same method (^13^), including a greater decrease in native and total thiol levels and a greater increase in disulfide, disulfide/native thiol, and disulfide/total thiol levels with increasing severity of DM complications and oxidative stress. These studies also investigated thiol/disulfide homeostasis in either prediabetes or in a single type of DM complication. Therefore, we designed a more comprehensive study involving prediabetes, DM without complications, and major DM complications to estimate oxidative stress levels by observing changes in thiol/disulfide homeostasis. Our results partially support the existing literature; for instance, the complexity of DM complications was associated with decreased levels of native and total thiol. However, our study differs from the others in terms of the trend in disulfide amount. Similar to our results, Altıparmak and cols. (^16^) have shown that levels of native thiol, total thiol, and disulfide are reduced in patients who undergo coronary angiography due to angina pectoris, and that this decrease is associated with the presence and severity of coronary artery disease.

Ates and cols. (^14^) suggested that the reason for the low native thiol in patients with prediabetes might be the increased proteinuria, which was not applicable to the present study, in our opinion. In contrast, Eren and cols. (^10^) emphasized a link between oxidative stress and hyperglycemia, and proposed that impaired thiol/disulfide homeostasis is a reason for diabetic nephropathy, which is supported by our findings.

In the present study, the diabetic foot group was the most affected in terms of alterations in thiol/disulfide parameters. This can be explained by the occurrence of poor vascularization and prolonged infection seen in foot ulcers. Furthermore, diabetic foot ulcer is a severe DM complication, as nontraumatic lower extremity amputations are most frequently caused by diabetic foot (^17^). Even though we found no studies in the literature comparing thiol/disulfide homeostasis in a healthy control group or DM-related complications with diabetic foot ulcers, some oxidative stress markers, such as 8-hydroxy-2’-deoxyguanosine (8-OHdG) and lipid peroxidase levels, have been assessed by researchers (^12^,^17^). İnce and cols. (^17^) found no significant difference in total thiol, native thiol, disulfide, and 8-OHdG levels between groups of patients with and without amputation, identified among patients with diabetic foot ulcers. Bolajoko and cols. (^12^) reported a significant increase in 8-OHdG and lipid peroxidase levels in a group of patients with diabetic foot ulcer compared with another group of controls without diabetes.

Native and total thiol levels have been shown to correlate negatively with plasma glucose levels, while the disulfide/native thiol and disulfide/total thiol ratios correlate positively, as observed in the study by Eren and cols. (^10^) - findings aligned with those in our study. The study by Gulpamuk and cols. (^15^) also found a significant negative correlation between native thiol and HbA1c levels in groups with and without proliferative diabetic retinopathy (r = -0.21 and p = 0.037; r = -0.040 and p = 0.004, respectively). Another study found a negative correlation between native thiol and total thiol versus age, BMI, baseline glucose on the OGTT, 120-minute glucose on the OGTT, and HbA1c level, although age, baseline glucose on the OGTT, 120-minute glucose on the OGTT, and HbA1c level correlated positively with disulfide level (^14^).

Thiol/disulfide homeostasis is a critical marker of oxidative stress in DM and its complications. Its clinical significance may include serving as a biomarker for oxidative stress, monitoring disease progression, acting as a therapeutic target to mitigate oxidative damage, predicting risk for developing complications, and evaluating the effectiveness of lifestyle and pharmacological interventions (^18^).

The present study, which suggests that thiol/disulfide homeostasis is altered due to DM complications, has several limitations. For instance, the DM complications were not classified into subgroups, and diabetic neuropathy was not included as one of the study groups. Another limitation was that the control group (Group 1) had a BMI value of 26.7 ± 6.1 kg/m^2^, categorized as overweight. The prevalence of overweight in Turkey is extremely high, with an overall estimated prevalence in men and women in 2010 of 37%, as reported by Erem in 2015 (^19^) . In the present study, 61.5% of the participants in Group 1 (n = 91) had a BMI above 25 kg/m^2^. If we excluded from Group 1 those participants who were overweight (BMI 26.7 ± 6.1 kg/m^2^), the group size would be substantially smaller. Therefore, we decided to include a control group of healthy volunteers (Group 1) based on their absence of DM or its complications. Additionally, since patients were on a wide range of medications and at different stages of disease, variations in medication or lifestyle factors were not included in this study, which is another limitation.

In conclusion, the present study compared thiol/disulfide homeostasis parameters in patients with prediabetes, diabetes mellitus without complications, and diabetes mellitus with different complications. The results of this study can be considered important in relating oxidative stress to different diabetes mellitus complications. The presence of diabetes mellitus complications was associated with a deterioration in thiol/disulfide homeostasis, significantly reducing levels of native and total thiol, as well as those of disulfide. Future studies could extend the present findings by including neuropathy and cardiovascular complications.

## Data Availability

the authors declare that no funds, grants, or other types of support were received during the preparation of this manuscript.

## Data availability:

datasets related to this article will be available upon request to the corresponding author.
